# Clinical Trial Acceptability and Preferences Among Patients with Corticobasal Syndrome

**DOI:** 10.1002/alz70859_107169

**Published:** 2025-12-26

**Authors:** Rebecca Snell, Brad F Boeve, Howard J. Rosen, Hilary W. Heuer, Adam L. Boxer, Lawren VandeVrede

**Affiliations:** ^1^ Memory and Aging Center, Weill Institute for Neurosciences, University of California, San Francisco, San Francisco, CA USA; ^2^ Department of Neurology, Mayo Clinic, Rochester, MN USA; ^3^ Department of Neurology, Memory and Aging Center, University of California San Francisco, San Francisco, CA USA

## Abstract

**Background:**

Corticobasal syndrome (CBS) is a neurodegenerative disease usually associated with accumulation of tau in the cortex and basal ganglia, leading to cognitive, movement, and behavioral symptoms that progressively impair function. Few clinical trials have ever been conducted in CBS, and perceptions and preferences about clinical trials in this population are largely unknown.

**Method:**

An online survey was conducted (February 2015 ‐ November 2020) among research participants recruited through a multisite observational study (ARTFL). Responses from patients with CBS were tabulated and reported below.

**Result:**

N=66 respondents had CBS as a primary or secondary phenotype (determined by study clinician), with 62% of surveys patient‐reported, and 38% completed by a caregiver/family member. Within CBS, 91% requested to be contacted about clinical trials. 2/6 decliners commented that disease severity precluded participation. 97% were willing to come in annually, 88% quarterly (3 months), 66% monthly, 43% weekly, and 17% would come in more than weekly; two patients could not travel due to disease severity. Comments included “we would live here for trials” and “whatever is needed.” Full day evaluations were acceptable to 91%, whereas 9% preferred a half‐day or less, although 65% would complete multi‐day evaluations spread over a week. Numerous remote and in person modes of assessment (e.g., surveys, examinations, cognitive testing, blood/saliva sampling, MRI, PET), were broadly acceptable by respondents (80‐93%), whereas remote activity monitoring (70%), lumbar puncture (68%), and remote video conferencing memory testing (67%) were less preferred, though still broadly acceptable. For distance willing to travel, 95% would travel 30 minutes, 91% one hour, 65% three hours, 54% five hours, and 37% would travel more than five hours or take a flight if needed. Travel reimbursement was very/extremely important for 40%, and unimportant for 26% (34% neutral). Feedback about clinical progression (81%), study results (74%), and an open‐label extension (68%) were factors identified as the highest importance to patients.

**Conclusion:**

Patients with CBS are eager to participate in clinical trials, even trials that are complex, far away, and have visits over multiple days. Travel reimbursement, providing feedback to patients, and access to experimental treatments were key considerations.

